# In Vitro Evaluation of Curcumin Encapsulation in Gum Arabic Dispersions under Different Environments

**DOI:** 10.3390/molecules27123855

**Published:** 2022-06-16

**Authors:** Dwi Hudiyanti, Muhammad Fuad Al Khafiz, Khairul Anam, Parsaoran Siahaan, Sherllyn Meida Christa

**Affiliations:** 1 Department of Chemistry, Faculty of Science and Mathematics, Diponegoro University, Jl. Prof. Soedarto, Semarang 50275, Indonesia; k.anam@live.undip.ac.id (K.A.); siahaan.parsaoran@live.undip.ac.id (P.S.); 2 Postgraduate Chemistry Program, Faculty of Science and Mathematics, Diponegoro University, Jl. Prof. Soedarto, Semarang 50275, Indonesia; queenfoe@gmail.com; 3 Chemistry Program, Faculty of Science and Mathematics, Diponegoro University, Jl. Prof. Soedarto, Semarang 50275, Indonesia; meidachrista@gmail.com

**Keywords:** gum Arabic, curcumin, drug delivery system, Simulated Intestinal Fluid, Simulated Gastric Fluid, encapsulation efficiency, loading capacity, antioxidant activity, release rate

## Abstract

Biopolymers, especially polysaccharides (e.g., gum Arabic), are widely applied as drug carriers in drug delivery systems due to their advantages. Curcumin, with high antioxidant ability but limited solubility and bioavailability in the body, can be encapsulated in gum Arabic to improve its solubility and bioavailability. When curcumin is encapsulated in gum Arabic, it is essential to understand how it works in various conditions. As a result, in Simulated Intestinal Fluid and Simulated Gastric Fluid conditions, we investigated the potential of gum Arabic as the drug carrier of curcumin. This study was conducted by varying the gum Arabic concentrations, i.e., 5, 10, 15, 20, 30, and 40%, to encapsulate 0.1 mg/mL of curcumin. Under both conditions, the greater the gum Arabic concentration, the greater the encapsulation efficiency and antioxidant activity of curcumin, but the worse the gum Arabic loading capacity. To achieve excellent encapsulation efficiency, loading capacity, and antioxidant activity, the data advises that 10% is the best feasible gum Arabic concentration. Regarding the antioxidant activity of curcumin, the findings imply that a high concentration of gum Arabic was effective, and the Simulated Intestinal Fluid brought an excellent surrounding compared to the Simulated Gastric Fluid solution. Moreover, the gum Arabic releases curcumin faster in the Simulated Gastric Fluid condition.

## 1. Introduction

Polymers are giant molecules with a high molecular weight (macromolecules) created by the covalent bonding of several smaller molecules or repeating units, called monomers. A natural polymer, found in plants, microorganisms, and animals, is one form of polymer based on its source of origin [1]. Natural polymers have several advantages over synthetic polymers, including homogeneous shapes and sizes, biodegradability, biocompatibility, non-toxicity, low cost, ease of modification, and accessibility [1,2,3,4]. Biopolymer is a natural polymer created directly by living organisms’ cells. It comprises bio-based monomer units covalently bound together to form bigger bio-based polymer molecules [5]. Polynucleotides (made of nucleotide monomers, e.g., DNA and RNA), polypeptides (composed of amino acid monomers, e.g., collagen), and polysaccharides (containing carbohydrate structures, e.g., starch, cellulose, and gum Arabic) are the three types of biopolymers [6]. Due to their biocompatibility, processability, and other benefits, natural polymers and biopolymers, mainly polysaccharides, are commonly used as drug carriers in drug delivery systems (DDS) [7]. By stabilizing the drug, localizing the drug’s action, and managing the release drug’s rate, time, and location, DDS is claimed to provide better therapeutic effects of the encapsulated drug at specific disease sites with low toxicological effects [8,9].

**Figure 1:**
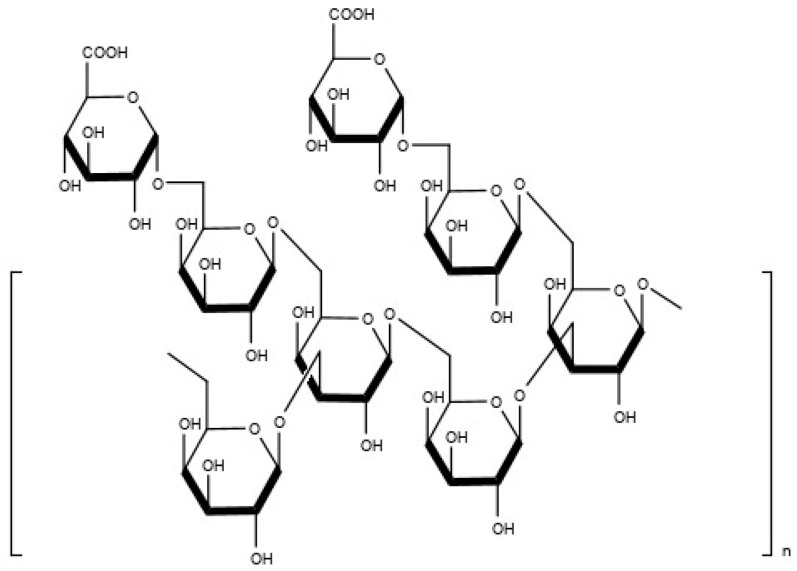
The structure of gum Arabic.

Gum Arabic (GA) in Figure 1 [10], also known as acacia gum, is the hardened sap of the Leguminosae family of *Acacia senegal* and *Acacia seyal* trees. It is a complex mixture of glycoproteins and polysaccharides, branched heteropolysaccharides that are either neutral or slightly acidic, light-orange or pale white, and water-soluble [11,12]. The GA structure’s mainframe comprises 1,3-linked β-d-galactopyranosyl units. At the same time, the side chains are made up of two to five 1,3-linked β-d-galactopyranosyl units that connect to the main chain via 1,6-linkages. Another study discovered that simple sugars such as d-galactose, l-arabinose, l-rhamnose, and d-glucuronic acid are also constituents of this heteropolysaccharide [13]. Because of biocompatibility, tastelessness, non-toxicity, and high-water solubility, GA is widely used as a drug carrier in DDS [14]. GA can also prevent aggregation as a drug carrier by forming a thick protective film around the encapsulated drug’s core material and acting as an emulsifier. Several studies have shown that using GA to encapsulate drugs or active compounds with antioxidant properties can improve the drug’s stability, encapsulation efficiency, and antioxidant capacity [15,16,17,18,19]. The ability of a mixture to scavenge free radicals by intervening in one of the three main steps of the oxidative process mediated by free radicals (i.e., initiation, propagation, and termination) is referred to as antioxidants [20,21].

Curcumin is the curcuminoid active compound found in turmeric (*Curcuma longa* L.) It has numerous health benefits, one of which is an antioxidant [22,23]. Curcumin’s unique reactive groups, which include two phenolic hydroxyl groups and an enol from a β-diketone moiety, are known to have potent free radical scavenging activity [24,25]. Due to their ability to directly react with free radicals and transform them into more stable or non-radical products, phenolic compounds with more than one hydroxyl group (–OH) are effective primary antioxidants [26,27]. Curcumin, poorly soluble in water (7.8 µg/mL), has low bioavailability in the body and a fast metabolism and excretion rate from the body’s system [28,29,30]. Curcumin is rapidly degraded in alkaline conditions (pH > 7) but degrades slowly in acidic conditions, implying that its decomposition is pH-dependent [31]. As a result, finding a suitable DDS is critical to overcoming the problem of delivering curcumin into the body for therapeutic use.

**Figure 2:**
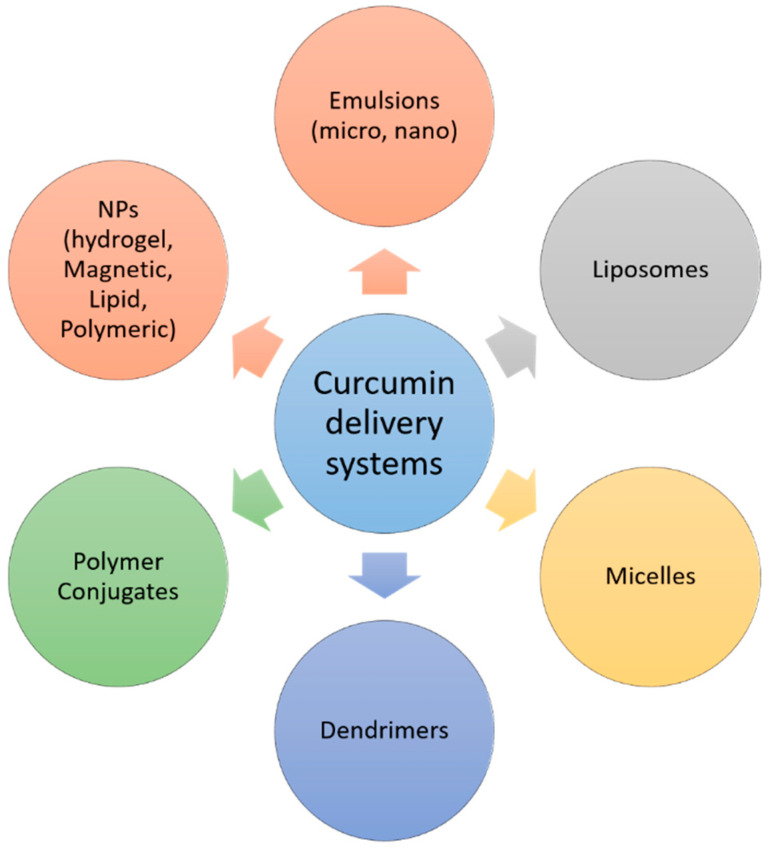
Representation of curcumin delivery systems.

Several studies have shown that liposomes as DDS can overcome curcumin’s weaknesses, allowing curcumin to be well encapsulated and its effectiveness in the body to improve [32,33,34,35,36]. Other materials of DDS, as depicted in Figure 2, such as dendrimers, micelles, and microemulsions, emulsions and nanoemulsions, solid lipid nanoparticles (SLNs), nanoparticles (NPs) including polymeric nanoparticles, magnetic nanoparticles, biopolymer nanoparticles, microgels, and hydrogel beads, have also been used to increase the solubility and bioavailability of curcumin so that it can be delivered into the body [36,37].

As mentioned above, GA has been widely used as the primary drug carrier or additional stabilizing material to improve the ability of encapsulated drugs when delivered into the body [14,16]. Therefore, this study aimed to investigate GA’s potential in encapsulating curcumin under two different oral drug delivery pathways, namely SIF and SGF solutions. Our new finding is that a 10% concentration of gum Arabic in both SIF and SGF solutions is the optimum concentration to achieve the optimal encapsulation efficiency of curcumin and the loading capacity of gum Arabic for curcumin.

## 2. Results and Discussion

Curcumin is a bioactive agent that is poorly soluble in water (7.8 µg/mL) [28], slightly improved under physiological pH conditions (0.0004 mg/mL) [36], easily soluble in organic solvents, including 96% ethanol (10 mg/mL) [38], and chemically unstable in gastric and intestinal environmental conditions [28]. Its decomposition depends on pH. Curcumin’s half-life at pH 3–6.5 is ~100–200 min, while at pH 7.2–8.0 it decreases significantly to only 1–9 min [37]. Research has shown that encapsulation using polymeric micelles, liposomes, or surfactant micelles can increase curcumin solubility [29,39]. In this research we establish that DDS using GA matrices, which are biopolymer, provide promising results in SIF and SGF solutions.

### 2.1. Encapsulation Efficiency, Loading Capacity, Release Rate, and Antioxidant Activity in SIF and SGF Solutions

Encapsulation efficiency (*EE*) is an important parameter to consider when evaluating the success of a DDS. The percentage of an encapsulated material (e.g., active ingredients, drugs, etc.) successfully entrapped into drug carriers following an encapsulation process for protection, absorption, delivery in the body, and controlled release is defined as *EE* [40,41]. Therefore, we investigated the *EE* of curcumin encapsulated in GA and expressed it as a percentage. It represents the amount of the drug encapsulated. In this study, the *EE* of curcumin was calculated indirectly by measuring the amount of the unencapsulated curcumin (*C_t_*) in the supernatant using UV-Vis spectrophotometer [42] and Equation (2).

**Figure 3:**
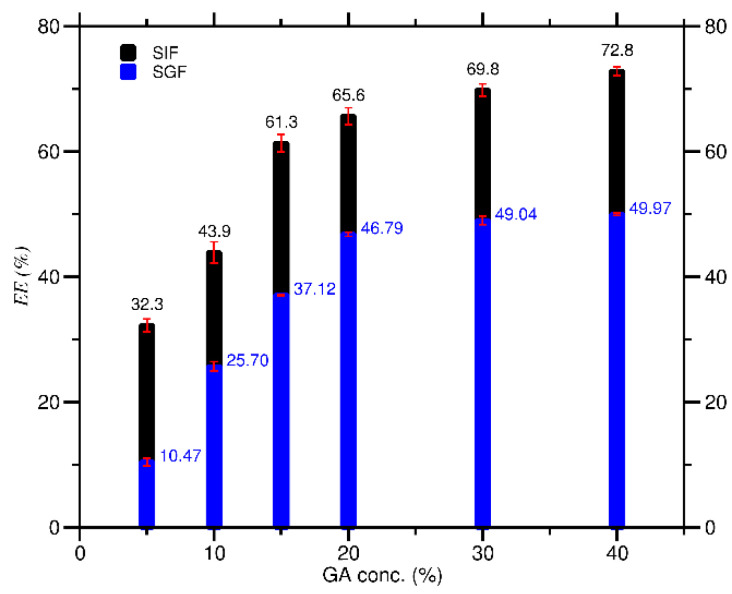
Encapsulation efficiency (*EE*) of curcumin at various *C_GA_* in SIF and SGF solutions.

The *EE* of curcumin increased as the concentration of GA (*C_GA_*) increased in both SIF and SGF conditions, as shown in Figure 3. *EE* grew rapidly at low *C_GA_* up to 20%, then relatively more slowly at higher *C_GA_* up to 40%. The data in Figure 3 also revealed that the *EE* for each *C_GA_* (5–40%) in SIF (range 32.3–72.8%) was more significant than that in SGF (range 10.47–49.97%). The higher the *EE* value obtained, the more curcumin was successfully encapsulated in GA. Thus, the *C_GA_* to get the highest *EE* for better DDS was 40% for both SIF (*EE* = 72.8%) and SGF (*EE* = 49.97%) conditions.

Loading capacity (*LC*) refers to a drug carrier’s ability to encapsulate a specific encapsulated material. The percentage of drugs incorporated within the drug carrier relative to the total mass of the drug carrier is referred to as LC. The drug carrier’s structural, physical, and chemical properties determine *LC* [41,43]. As shown in Equation (3), *LC* in this study can be calculated by dividing the total concentration of successfully encapsulated curcumin (*C*_0_–*C_t_*) by the total concentration of GA (*C_GA_*). The higher the *LC* value of GA, the more curcumin was successfully encapsulated. This indicates that the best potential of GA as a drug carrier in DDS (composed of the drug carrier and the encapsulated material) can be obtained at this *C_GA_* because curcumin can be maximally encapsulated [44,45].

**Figure 4:**
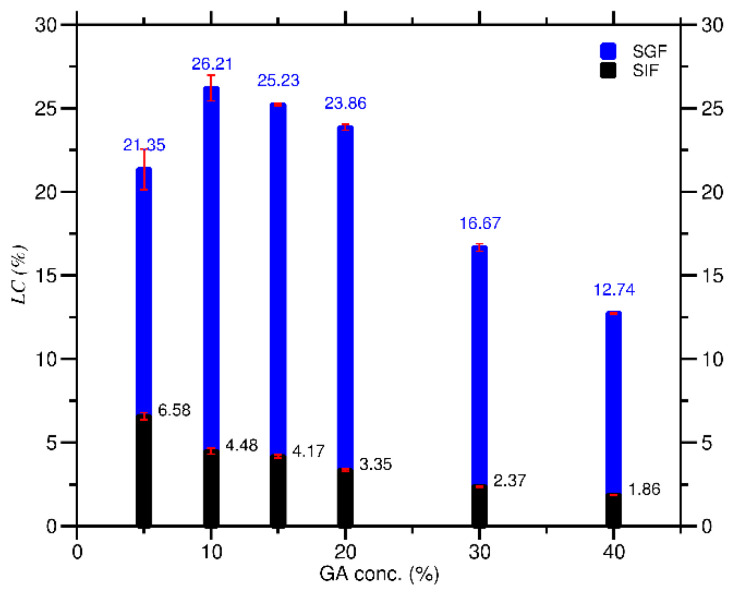
Loading capacity (*LC*) of GA at various *C_GA_* in SIF and SGF solutions.

As shown in Figure 4, the *LC* decreased as the *C_GA_* increased in SIF and SGF conditions except for GA in SGF with a 5% to 10% concentration. The *LC* between these concentrations increased by 4.86%, from 21.35% to 26.21%. The data in Figure 4 also revealed that the *LC* for each *C_GA_* (5–40%) in SGF (range 26.21–12.74%) was greater than that in SIF (range 6.58–1.86%). The lowering of the loading capacity of GA is assumed because the carboxylic group in GA has been wholly ionized to COO^−^. The formation of this charge creates a repulsion force between the acid groups of GA, resulting in destabilization of the GA structure and a decrease in *LC* [16]. The *C_GA_* for obtaining the highest *LC* value for DDS was 5% for SIF (6.58%) and 10% for SGF (26.21%) conditions.

The rate of drug release (*RR*) from a DDS to the desired target tissues is a critical property associated with a drug’s therapeutic activity in the body [46]. Hence, we investigated the release rate of curcumin encapsulated in GA. A controlled rate of release of a DDS is a delivery form in which the drug is released at a predetermined rate based on the desired therapeutic concentration and the drug’s pharmacokinetic properties [47]. Because the release rate has been determined, the medication delivered can have a long lifetime ranging from days to months, with minimal side effects on the body.

**Figure 5:**
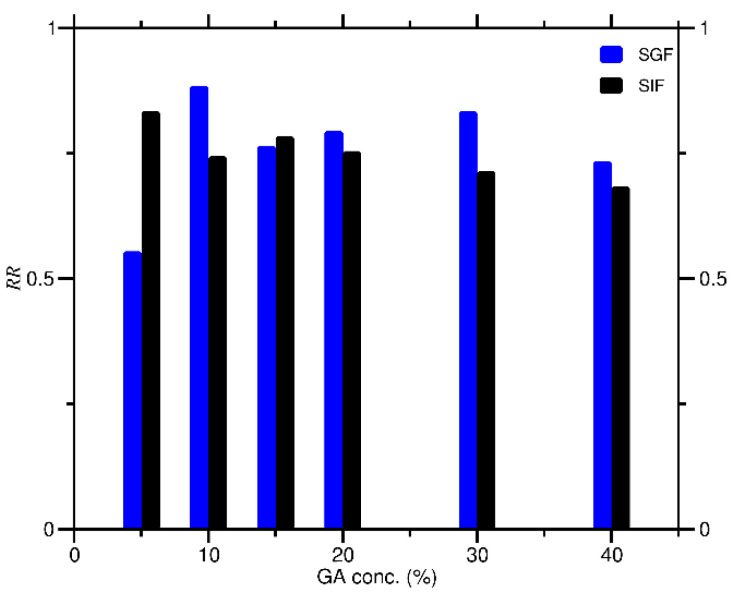
Release rate (*RR*) of curcumin encapsulated in GA at various *C_GA_* in SIF and SGF solutions.

The *RR* of curcumin encapsulated in GA varied as the *C_GA_* increased in SIF and SGF conditions, as shown in Figure 5. The *RR* of curcumin encapsulated in GA dispersed in SIF was higher than that of SGF at 5% and 15% of *C_GA_*, respectively. However, at other *C_GA_* (10%, 20%, 30%, and 40%), the *RR* in SGF was higher than that in SIF. The observation of the *RR* for 12 days revealed that under SIF conditions, curcumin encapsulated in GA lasted the longest at 40% of *C_GA_*, while, under SGF conditions, curcumin encapsulated in GA lasted the longest at 10% of *C_GA_*. In general, curcumin would be released faster in the SGF compared to the SIF condition.

We estimate the mechanism of curcumin encapsulation in GA occurs due to non-covalent interactions between the –COOH group of GA and the –OH group of curcumin to form hydrogen bonds. The formation of hydrogen bonds affects both the encapsulation of curcumin in GA and the release of curcumin from GA. This hydrogen bond formation also explains why the *EE* and *LC* of GA to encapsulate curcumin are higher than that of tocopherol [16]. Curcumin has additional OH groups compared to tocopherol, so more hydrogen bonds are formed during the encapsulation process. Further studies are still needed to confirm the hydrogen bonding formation such as using X-ray Diffraction (XRD), Differential Scanning Calorimetry (DSC), or computational studies. 

Antioxidant activity (*IR*) is defined as limiting or inhibiting the oxidation of nutrients (particularly lipids and proteins) by preventing oxidative chain reactions from occurring. We used the DPPH scavenging activity assay to assess the *IR* of curcumin encapsulated in GA, as shown in Equation (4). If the scavenging activity of DPPH is high, the value of *IR* will be increased. If the value of DPPH is higher, it means that the amount of antioxidant compounds in the related drug (e.g., curcumin) is smaller [21,32,48]. The lower the number of antioxidant compounds required to obtain a high value of IR, the better the compound’s ability to defend against free radicals in its role as an antioxidant [49,50,51].

**Figure 6:**
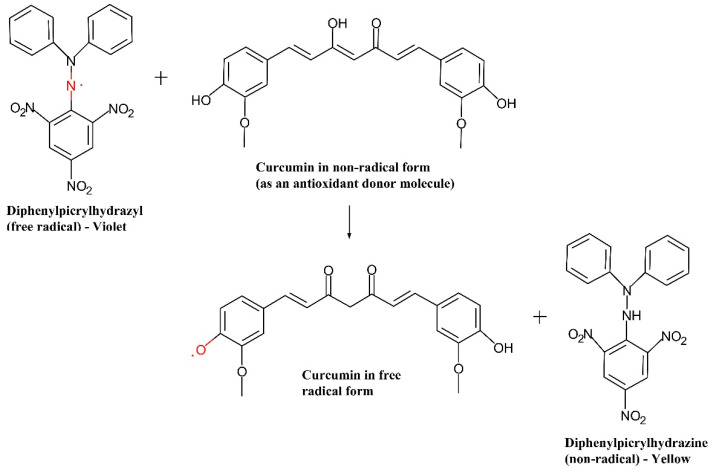
Antioxidant reaction mechanism of radical form DPPH with curcumin.

When the odd electron from the nitrogen atom in the radical form of DPPH accepts a hydrogen atom from the antioxidant, it undergoes reduction. It forms the corresponding hydrazine or non-radical form of DPPH [48,52]. Overall, the DPPH molecule is classified as a stable free radical due to the delocalization of the spare electron across the molecule, which prevents the molecule from dimerizing like most other free radicals. The presence of electron delocalization results in a deep violet color, with absorption in ethanol solution at around 515–517 nm. When the DPPH solution is mixed with an antioxidant compound that donates a hydrogen atom, such as curcumin, it loses its deep violet color (becomes colorless or pale yellow in color), as shown in Figure 6 [21,53,54].

Equation (1) depicts the primary reaction in which the DPPH radical is *Z*•, whose activity will be suppressed by *AH* as an antioxidant donor molecule, *ZH* is the reduced form of DPPH (non-radical), and *A*• is the antioxidant donor molecule’s free radical form [21,55].

(1) Z•+AH=A•+ZH 

In terms of the number of electrons taken up, the decolorization in the DPPH molecule that reacts with antioxidants is stoichiometric. DPPH can respond with the entire sample, even if the antioxidants are weak. Therefore, the DPPH free radical scavenging assay method is widely used to assess a compound’s ability to act as a free radical scavenger or hydrogen donor and its antioxidant activity (*IR*) [55,56].

**Figure 7:**
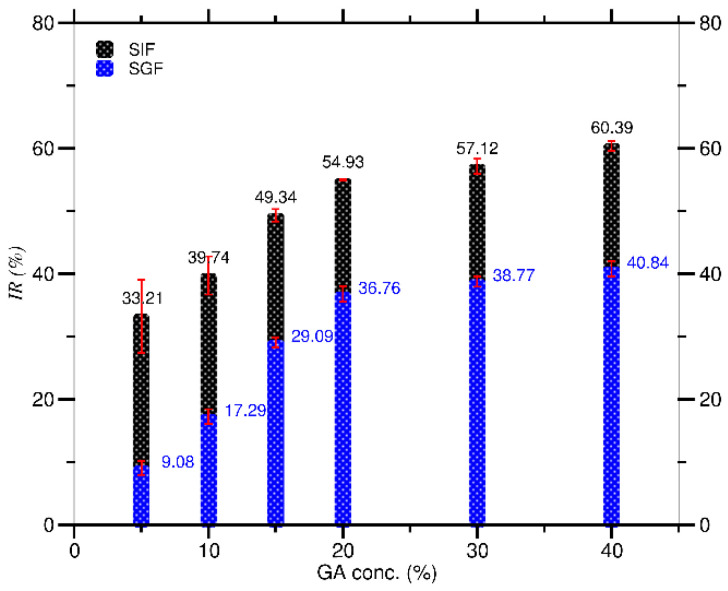
Antioxidant activity (*IR*) at various *C_GA_* in SIF and SGF solutions.

In both SIF and SGF conditions, as shown in Figure 7, the *IR* increased as the *C_GA_* increased. The *IR* in SIF (range 33.21–60.39%) was higher than in SGF (range 9.08–40.84%), indicating that curcumin’s antioxidant activity was better in SIF than in SGF conditions. This is due to the nature of curcumin, which degrades quickly in alkaline but slowly in acidic conditions [31], resulting in a decrease in the amount of undegraded curcumin as an antioxidant compound that will react with DPPH. This decrease in the curcumin results in a high value of DPPH scavenging activity, which directly impacts the *IR* value in SIF rather than SGF. Furthermore, curcumin in SIF appears in the enolate form of the heptadienone chain (an electron donor), whereas curcumin in SGF appears in a protonated form (a hydrogen donor). Because only hydrogen donors can react with DPPH, SIF has a higher *IR* value than SGF [32,57]. The higher the *IR* value at a high *C_GA_*, the better curcumin’s antioxidant performance against free radicals. It is also aided by GA, which has antioxidant activity [17,19]. Therefore, the *C_GA_* for obtaining the highest antioxidant activity of curcumin was 40% for both dispersions in SIF (60.39%) and SGF (40.84%) conditions.

### 2.2. Optimum Encapsulation Efficiency and Loading Capacity in SIF and SGF Solutions

The *EE* of the encapsulated material and the *LC* of the drug carrier are both parameters that are closely related to the ability of a DDS to encapsulate a drug for delivery to specific sites in the body. This study will compare the *EE* of curcumin, and the *LC* of GA dispersed in SIF and SGF to determine the optimum value between these two parameters. 

**Figure 8:**
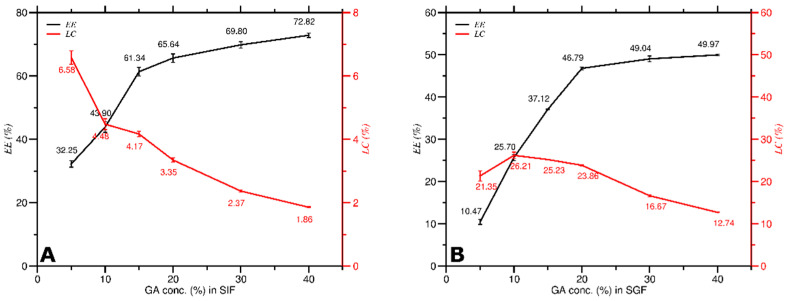
Encapsulation efficiency of curcumin vs. loading capacity of GA at various *C_GA_* in, (**A**) SIF solution; (**B**) SGF solution.

*EE* increased while *LC* decreased as *C_GA_* increased in SIF and SGF solutions as shown in Figure 8. Furthermore, Equation (2) demonstrates that the *EE* value was directly proportional to both the encapsulated curcumin concentration and the *C_GA_*, whereas Equation (3) demonstrates that the *LC* value was directly proportional to the encapsulated curcumin concentration and inversely proportional to the *C_GA_*. Therefore, *EE* is inversely proportional to *LC*, consistent with the results. The higher the *C_GA_* used, the easier it was for curcumin to be encapsulated (higher value of *EE*), but it further reduced the space of GA to encapsulate curcumin again (lower value of *LC*).

The *EE* ranged from 32.25–72.82% for *C_GA_* dispersed in SIF, and the *LC* ranged from 6.58–1.86%. The *EE* of curcumin increased significantly at 5–15% of *C_GA_*, whereas the *LC* of GA decreased significantly at 5% of *C_GA_* as shown in Figure 8A. The *EE* ranged from 10.47% to 49.97% for *C_GA_* dispersed in SGF, and the *LC* ranged from 21.35 to 12.74%. The *EE* of curcumin increased significantly at 5–20% of *C_GA_*, whereas the *LC* of GA decreased substantially at 20% of *C_GA_* as shown in Figure 8B. The data recommend that 10% is the optimum *C_GA_* to obtain the optimum *EE* of curcumin and *LC* of GA of the encapsulation process in SIF and SGF conditions.

### 2.3. Relationship of Encapsulation Efficiency and Antioxidant Activity of Curcumin in SIF and SGF Solutions

These two parameters relate to the amount of curcumin encapsulated in GA (*EE*) and the ability of curcumin to act as an antioxidant compound (*IR*) in different pH environments.

**Figure 9:**
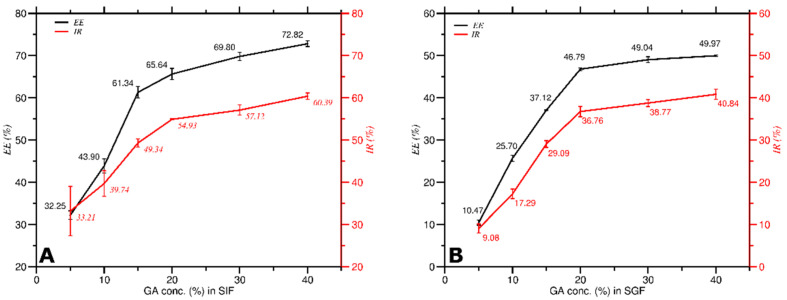
Encapsulation efficiency of curcumin vs. antioxidant activity at various *C_GA_* in, (**A**) SIF solution; (**B**) SGF solution.

Both *EE* and *IR* increased as *C_GA_* increased in SIF and SGF conditions as shown in Figure 9. According to Equation (2), the *EE* value was directly proportional to both the encapsulated curcumin concentration and the *C_GA_*, whereas Equation (4) shows that the *IR* value was directly proportional only to the antioxidant activity of DPPH radical that reacts with GA-Curcumin to form non-radical DPPH and inversely proportional to the antioxidant activity of only DPPH radicals. Therefore, the results in which *EE* is directly proportional to *IR* align with the theoretical suggestion. The higher the *C_GA_*, the more curcumin was successfully encapsulated in GA (higher value of *EE*) and the higher curcumin’s ability as an antioxidant to ward off free radicals (higher value of *IR*).

The *EE* and *IR* progression values in both SIF (Figure 9A) and SGF (Figure 9B) showed that SGF gave a higher increment than SIF. This suggests that regarding *EE* and *IR*, SGF gave better environmental conditions for encapsulation of curcumin in GA. Furthermore, the results indicate that the highest increase in *EE* and *IR* in SIF and SGF occurred between 5% and 20% of *C_GA_*.

### 2.4. Relationship of Loading Capacity and Antioxidant Activity of Curcumin in SIF and SGF Solutions

These parameters relate to the ability of GA as a drug carrier for curcumin (*LC*) and the power of GA to stabilize further and enhance curcumin’s ability as an antioxidant compound (*IR*) in two different pH surroundings.

**Figure 10:**
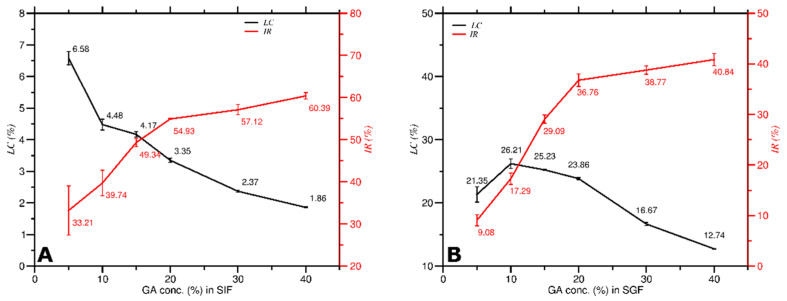
Loading capacity of GA vs. antioxidant activity at various *C_GA_* in, (**A**) SIF solution; (**B**) SGF solution.

As the *C_GA_* increased in both SIF and SGF as shown in Figure 10, *LC* decreased while *IR* increased, implying that *LC* was inversely proportional to *IR*. The results suggest that a high amount of curcumin loading in GA was not adequate concerning the antioxidant activity of curcumin.

In SIF (Figure 10A), the *LC* ranged from 6.58 to 1.86%, and the *IR* ranged from 33.21 to 60.39%. The *LC* of GA decreased significantly at 5% of *C_GA_*, whereas the *IR* increased significantly at 5–20% of *C_GA_*. In SGF (Figure 10B), the *LC* ranged from 21.35 to 12.74%, and the *IR* ranged from 9.08 to 40.84%. The *LC* of GA decreased significantly at 20% of *C_GA_*, while the *IR* increased significantly at 5–20% of *C_GA_*. These outcomes propose that the optimum *C_GA_* for obtaining the optimum *LC* of GA and *IR* of curcumin is around 10% to 20% in SIF and SGF conditions. The SGF provided a better atmosphere than the SIF solution regarding the antioxidant activity.

This study provides several parameters that feature the encapsulation of curcumin in GA, namely *EE*, *LC*, and release of curcumin encapsulation in GA. To understand more about the curcumin delivery system using GA, further study is necessary to analyze other physicochemical characteristics of the dispersion, including the particles’ size, shape, and surface charge. These parameters are crucial for a successful delivery system.

## 3. Materials and Methods

### 3.1. Materials

The materials used were gum Arabic, curcumin, Na_2_HPO_4_·2H_2_O (0.05 M), NaH_2_PO_4_·2H_2_O (0.05 M), HCl (37%), NaCl, NaOH, ethanol, DPPH solution (40 µg/mL), and demineralized water.

### 3.2. Methods

#### 3.2.1. Preparation of Simulated Intestinal Fluid (SIF)

A solution of 0.05 M was prepared from 7.5 g of Na_2_HPO_4_·2H_2_O in 500 mL of demineralized water. Another solution of 0.05 M was also prepared from 3.9 g of NaH_2_PO_4_·2H_2_O in 500 mL of demineralized water. A mixture of 9.5 mL of NaH_2_PO_4_·2H_2_O (0.05 M) and 40.5 mL of Na_2_HPO_4_·2H_2_O (0.05 M) was prepared and diluted into 100 mL. The pH was adjusted to 7.4.

#### 3.2.2. Preparation of Simulated Gastric Fluid (SGF)

In 800 mL of demineralized water, about 2 g of NaCl was dissolved. Drop by drop, a total of 4.5 mL of 37% HCl solution was added into the NaCl solution, followed by demineralized water until the volume reached 1 L. The pH of the solution was tuned to 1.2.

#### 3.2.3. Curcumin Encapsulation in Gum Arabic (GA)

A series of GA dispersions with concentrations (*C_GA_*) of 5%, 10%, 15%, 20%, 30%, and 40% (w/v) were prepared in 100 mL of chloroform/methanol (9/1, v/v). Curcumin, at a concentration of up to 0.1 mg/mL per GA dispersion, was first dissolved in a small amount (a few drops) of ethanol before being added to each GA dispersion, then stirred for 10 min. The ethanol facilitates the mixing of the curcumin with the GA dispersion. In a test tube, 10 mL of GA-curcumin dispersion was streamed with nitrogen gas until a thin layer remained at the bottom. After that, 10 mL of the SIF solution was added to the test tube containing a thin layer, and the freeze-thawing process was continued in the test tube. The freeze-thawing cycle adapted from Hudiyanti’s research [32,58,59,60] was carried out by cooling it at 4 °C and heating it at 45 °C repeatedly until the thin layer was completely dissolved. Then it was sonicated for 30 min at 27 °C. This procedure for repeated for each GA concentration in both SIF and SGF solutions. Next, 1 mL of sonicated GA-curcumin dispersion was dissolved in 5 mL of ethanol (w/v), then centrifuged at 3461× *g* for 40 min until two layers were formed in the test tube. The watery top layer (supernatant) containing unencapsulated curcumin was separated for analysis on *LC* and *EE*. The thick bottom layer (GA residue) was stored at −18 °C until it was reused for analysis of *IR* and *RR*. The sink condition was maintained at 30 °C for all compositions.

#### 3.2.4. Curcumin Encapsulation Efficiency (*EE*) and GA Loading Capacity (*LC*)

The *EE* of curcumin and *LC* of GA were evaluated based on the concentration of unencapsulated curcumin in the supernatant. The unencapsulated curcumin in the supernatant, which had previously been dissolved in ethanol before the centrifugation process, was then analyzed using a UV-Vis spectrophotometer. The concentration of unencapsulated curcumin (*C_t_*) was analyzed at a wavelength of 426 nm. The *EE* of curcumin was calculated with Equation (2), while the *LC* of GA was calculated with Equation (3) [16].

(2) $$EE=\left[1-\left(\frac{C_{t}}{C_{0}}\right)\right]\times 100\%$$

(3) $$LC=\left(\frac{C_{0}-C_{t}}{C_{GA}}\right)\times 100\%$$

#### 3.2.5. Analysis on Curcumin Release Rate (*RR*)

The *RR* of curcumin was determined using the concentration of curcumin released from GA during storage. The GA residue obtained from the previous encapsulation procedure was dispersed in the buffer solution, i.e., SIF and SGF solutions (1/5, w/v), and stored in an incubator at 4 °C. Curcumin release from GA into the buffer solution was monitored for 12 days. Each dispersion was homogenized using an ultrasonic homogenizer for 5 min, followed by centrifugation at 4500 rpm for 15 min. The dispersion was then allowed to settle and form two layers. The absorbance of the supernatant separated from the GA residue was measured at a wavelength of 426 nm. This procedure was repeated for each GA dispersion.

#### 3.2.6. Analysis of DPPH Free Radical Scavenging Assay (Antioxidant Activity, *IR*)

The Blois method [55] was used to perform the 1-Diphenyl-2-picrylhydrazyl (DPPH) free radical scavenging assay, in which 1 mL of each GA-Curcumin dispersion was mixed with 3 mL of DPPH (40 µg/mL) solution. The mixture was then incubated for 30 min at room temperature without exposure to light. The absorbance of the mixture was measured with a UV-Vis spectrophotometer at a maximum wavelength (λ_max_) of 515 nm. The DPPH antioxidant activity (*IR*) was calculated using Equation (4) as follows:

(4) $$IR=\left(\frac{A_{0}-A_{1}}{A_{0}}\right)\times 100\%$$

where *A*_0_ is the absorbance of the DPPH solution without the addition of GA-Curcumin dispersion and *A*_1_ is the absorbance of the DPPH solution with the addition of GA-Curcumin dispersion after 30 min of incubation [32].

#### Statistical Analysis

All data presented in this article were acquired in triplicate. The data were presented as mean ± standard deviation (SD).

## 4. Conclusions

We have successfully encapsulated curcumin in gum Arabic dispersions in the SIF and SGF solutions. The results give a satisfactory outcome regarding the potency of gum Arabic for the encapsulation of curcumin in both environments. The higher the gum Arabic concentration, the higher the encapsulation efficiency and antioxidant activity of curcumin, but the lower the gum Arabic loading capacity. The data propose that 10% is the best possible gum Arabic concentration to achieve the optimal encapsulation efficiency of curcumin and the loading capacity of gum Arabic for curcumin. Regarding the antioxidant activity of curcumin, the results indicate that an excessive concentration of gum Arabic was effective, and the SIF delivered a superior milieu than the SGF solution. Moreover, the gum Arabic would release curcumin more quickly in the SGF setting.

## Data Availability

All data generated or analysed during this study are included in this published article.

## Sample Availability

Samples of the compounds are available from the authors.
